# PCSK9-Targeting Drugs and Gender: Are There Any Differences?

**DOI:** 10.3390/jcm14134469

**Published:** 2025-06-24

**Authors:** Viola Liberati, Giulia Guidotti, Andrea Sorrentino, Margherita Slanzi, Elena Lotti, Felice Crudele, Angela Rogolino, Francesco Alfano, Betti Giusti, Anna Maria Gori, Martina Berteotti, Rossella Marcucci

**Affiliations:** 1 Department of Experimental and Clinical Medicine, University of Florence, 50134 Florence, Italy; viola.liberati@unifi.it (V.L.); giulia.guidotti@unifi.it (G.G.); margherita.slanzi@edu.unifi.it (M.S.); francesco.alfano@unifi.it (F.A.); betti.giusti@unifi.it (B.G.); annamaria.gori@unifi.it (A.M.G.); martina.berteotti@unifi.it (M.B.); 2 Atherothrombotic Diseases, “Careggi” University Hospital, 50134 Florence, Italy; lottie@aou-careggi.toscana.it (E.L.); crudelef@aou-careggi.toscana.it (F.C.); rogolinoa@aou-careggi.toscana.it (A.R.)

**Keywords:** PCSK9 inhibitors, female sex, sex differences, real-world data, hypercholesterolemia, high-risk patients, HeFH

## Abstract

**Background:** Atherosclerotic cardiovascular disease (ASCVD) is often perceived as a male-dominant condition, yet recent European data show that more women live with and die from it. Gender disparities have been reported in the management of dyslipidemia, with women less likely to receive high-intensity lipid-lowering therapy and to reach low-density lipoprotein cholesterol (LDL-C) goals. This study aimed to assess sex-specific differences in response to and tolerance of PCSK9-targeted therapies—monoclonal antibodies (evolocumab, alirocumab) and small interfering RNA (inclisiran)—as well as LDL-C goal attainment according to current ESC guidelines. **Methods:** We conducted a prospective registry of patients initiating PCSK9-targeted therapy at a specialized lipid center between April 2018 and June 2024. Baseline lipid profiles were recorded and monitored over follow-up. **Results:** Of the 341 patients, 122 (35.8%) were women and 219 (64.2%) were men, with a mean age of 66.4 ± 12.6 years for the women and 63.9 ± 11.8 years for the men. The women more frequently had heterozygous familial hypercholesterolemia (HeFH) (61.5% vs. 38.4%, *p* < 0.001) and a lower prevalence of previous cardiovascular events compared to the men (62.3% vs. 84.5%, *p* < 0.001). A higher proportion of the women were classified as high cardiovascular risk compared to the men (37.7% vs. 15.5%, *p* < 0.001). Risk categories were assigned according to ESC guidelines, with LDL-C targets of <70 mg/dL for high-risk patients and <55 mg/dL for very high risk patients, along with a ≥50% LDL-C reduction for both categories. In the very high risk group, fewer women achieved LDL-C targets at the first two follow-up visits (first follow-up: 50.0% vs. 76.6%, *p* = 0.008; second follow-up: 55.3% vs. 68.1%, *p* = 0.049). Although treatment prescription and tolerance were similar between sexes, women showed smaller LDL-C reductions at the first follow-up (51.7 ± 23.9% vs. 57.3 ± 24.9%, *p* = 0.044). **Conclusions:** PCSK9-targeted therapies were effective in both sexes at third follow-up, although women showed a tendency toward a delayed response and lower target attainment, indicating the potential need for more personalized management strategies.

## 1. Introduction

In 1991, the “female issue” in medicine was first addressed by Dr. Bernardine Patrice Healy, an American cardiologist, in an editorial published in the *New England Journal of Medicine* titled “The Yentl Syndrome.” The article highlighted sex-based disparities in the management of coronary artery disease, emphasizing a higher rate of diagnostic errors in women and a lower likelihood of revascularization procedures being performed [1,2,3]. Since then, numerous studies have emerged, shedding light on areas where the clinical management of women’s health can be improved [4,5,6,7,8].

Cardiovascular diseases are currently the leading cause of death among women [9]. However, perception of cardiovascular risk remains low, leading to underdiagnosis and delayed treatment, especially as many believe women, particularly those of reproductive age, are protected by estrogen [10,11]. Gaps in medical knowledge have highlighted gender-specific risk factors that are often overlooked and the perception of cardiovascular risk among women remains low, leading to underdiagnosis, delayed treatment, and inadequate preventive measures [12,13].

LDL cholesterol (LDL-C) is one of the main modifiable cardiovascular risk (CV) factors, since the accumulation of LDL-C and other apo-B-containing lipoproteins is a key event in the initiation and progression of the atherosclerotic process [14,15]. Target LDL-C levels have been progressively reduced, but the gap between guidelines and actual LDL-C levels achieved in practice persists, especially in high-risk patients [16,17,18].

Research has revealed a sex disparity in the management of dyslipidemia, with women being significantly more likely to fail to achieve LDL-C targets and less likely to be prescribed high-intensity lipid-lowering therapies in response to elevated LDL-C levels. Women are also more prone to experiencing statin-associated adverse effects, which can negatively influence adherence and treatment escalation. Furthermore, women remain under-represented in most lipid-lowering clinical trials, limiting the statistical power to detect true sex-specific differences and leading to therapeutic uncertainty in female patients [19,20,21].

Among the available therapeutic strategies, proprotein convertase subtilisin/kexin type 9 monoclonal antibodies (PCSK9mAb) have become an essential treatment option for patients with high cardiovascular risk, particularly for those who do not reach LDL-C targets despite maximally tolerated statin therapy. PCSK9mAb treatments were prescribed according to the ESC Guidelines’ recommendations and the Italian Medicines Agency (AIFA) reimbursement criteria. Specifically, patients aged 18 to 80 years old received PCSK9mAb treatment if they had either of the following:

1. Asymptomatic heterozygous familial hypercholesterolemia (HeFH) and LDL-C ≥ 130 mg/dL despite at least 6 months of high-potency statins at the maximum tolerated dose combined with ezetimibe, or those with statin and/or ezetimibe intolerance.
2. Known ASCVD (atherosclerotic cardiovascular disease) and LDL-C ≥ 70 mg/dL despite 6 months of high-potency statins combined with ezetimibe, or those with recent myocardial infarction (within the last 12 months) or multiple cardiovascular events, or statin and/or ezetimibe intolerance [22].

Trials have demonstrated that PCSK9 inhibitors—both antibodies and inclisiran—can achieve about a 50% reduction in LDL-C, resulting in improved cardiovascular prognosis and reduced mortality [23,24]. However, even with the integration of PCSK9 inhibitors, which enable approximately half of treated patients to achieve lipid targets, sex differences seem to persist [25,26,27].

Current evidence on sex-related differences in the therapeutic management of patients receiving these novel lipid-lowering agents remains limited. Despite similar indications for the use of PCSK9 inhibitors, women generally exhibit higher mean LDL-C levels compared to men. Moreover, men are more frequently represented among patients treated with monoclonal antibodies, and a lower proportion of women reach the LDL-C targets recommended by clinical guidelines when treated with evolocumab or alirocumab [26]. In contrast, data on sex-specific outcomes with inclisiran are still scarce.

The aim of this analysis was to explore potential sex differences in response to and tolerance of novel PCSK9-targeting therapies (both monoclonal antibodies—evolocumab and alirocumab—and siRNA—inclisiran) and to investigate adherence to LDL-C targets in accordance with the latest ESC guidelines [17].

## 2. Materials and Methods

This observational, prospective, single-center study included patients from the Atherothrombotic Diseases Unit of Careggi University Hospital in Florence (Italy) who initiated therapy with evolocumab, alirocumab, or inclisiran between April 2018 and June 2024.

PCSK9 inhibitors were prescribed according to the reimbursement criteria set by the Italian Medicines Agency (AIFA). Monoclonal anti-PCSK9 antibodies were self-administered, while inclisiran was administered by healthcare personnel in an outpatient setting. Evolocumab and alirocumab were given biweekly or monthly, while inclisiran followed an induction phase, with doses given three months apart, then a maintenance phase with the third dose administered after six months.

The decision to prescribe this medication was made as part of the department’s routine clinical practice and was independent of the decision to include patients in the study.

All participants provided written informed consent prior to enrollment. The study was conducted in accordance with the Declaration of Helsinki, and ethical approval was obtained from the Ethics Committee of Careggi University Hospital.

At treatment initiation, all patients underwent a standardized clinical-functional evaluation, including demographic data, cardiovascular risk factors, dyslipidemia type, prior atherosclerotic events and interventions, comorbidities, cardiovascular risk level (high and very high), background lipid-lowering therapy, statin intolerance, and type of anti-PCSK9 used. Statin intolerance was determined from reported symptoms or abnormal lab results (e.g., elevated CPK or transaminases); this condition may be either complete (intolerance to all statins at any dose) or partial (tolerance only to reduced doses or intermittent regimens). HeFH was diagnosed using the Dutch Lipid Clinical Network Score for FH (DLCNS), with or without genetic testing. The lipid profile was assessed prior to the initiation of anti-PCSK9 therapy and monitored at scheduled follow-up visits. For patients receiving evolocumab or alirocumab, follow-up visits occurred at 6, 12, and 18 months after treatment initiation, while for those on inclisiran, visits were scheduled at 3, 9 and 15 months. Lipid profiles were measured at each of these visits. Only patients with at least one post-baseline LDL-C measurement were included in the final analysis. The primary aim of this study was to evaluate potential sex differences in response to and tolerance of treatment with PCSK9 inhibitors and to assess possible differences in achieving the therapeutic targets recommended by the latest ESC guidelines on dyslipidemias [22]. Therefore, we assessed the proportion of women and men achieving an LDL-C reduction of ≥50% from baseline plus their CV risk-class-specific LDL-C targets: <55 mg/dL for very high risk CV patients and <70 mg/dL for high-risk CV patients. Furthermore, we sought to identify predictive factors that may either hinder or facilitate the attainment of the LDL-C levels indicated by the guidelines in both sexes. As a complementary objective, we performed a stratified analysis to explore sex-specific differences in LDL-C response across the different PCSK9-targeting agents.

### Statistical Analysis

Statistical analyses were conducted using IBM SPSS Statistics for Macintosh, version 27.0 (IBM Corp., Armonk, NY, USA). Categorical variables were reported as frequencies and percentages, while continuous variables were expressed as mean ± standard deviation (SD) or median with interquartile range (Q1, Q3), depending on data distribution. Normality was assessed using the Shapiro–Wilk test.

Univariate comparisons between male and female patients were initially performed using the chi-squared test for categorical variables, the unpaired *t*-test for normally distributed continuous variables, and the Wilcoxon rank-sum test for non-normally distributed variables. This descriptive approach was adopted to reflect real-world differences between sexes at baseline.

To identify independent predictors of LDL-C-target achievement, univariate Poisson regression analyses were first performed. Variables found to be significantly associated with the outcome (*p* < 0.10) were included in a multivariate Poisson regression model with robust standard errors. The variables considered included sex, age, baseline LDL-C, cardiovascular risk category, type of dyslipidemia, statin intolerance (complete or partial), type of PCSK9 inhibitor, and presence of prior ASCVD. Sex was retained in the model regardless of statistical significance, as a variable of primary interest.

All statistical tests were two-tailed, and a *p*-value < 0.05 was considered statistically significant.

## 3. Results

The study included 341 patients, consisting of 122 women (35.8%) and 219 men (64.2%), with a mean age of 66.4 ± 12.6 years for the women and 63.9 ± 11.8 years for the men.

A notable sex difference was observed in the classification of cardiovascular risk: a higher proportion of the women were categorized as high-risk (37.7% vs. 15.5%, *p* < 0.001), whereas more of the men were classified as very high risk (84.5% vs. 62.3%, *p* < 0.001). The women had a greater prevalence of heterozygous familial hypercholesterolemia (61.5% vs. 38.4%, *p* < 0.001) and a lower smoking history (31.1% vs. 47%, *p* = 0.004), while the men exhibited a higher prevalence of coronary artery disease (75.3% vs. 40.2%, *p* < 0.001) and underwent more percutaneous coronary interventions (65.3% vs. 32.8%, *p* < 0.001). No significant sex differences were found in rates of coronary artery bypass grafting, cerebrovascular events, or peripheral artery disease.

Regarding background therapies, a significant sex difference was observed only in the cohort receiving statin–ezetimibe combination therapy (men: 52.5% vs. women: 30.3%, *p* = 0.041). Statin intolerance showed a significant difference between sexes (men: 43.8% vs. women: 55.7%, *p* = 0.035).

Of the 341 patients, 136 (39.9%) received evolocumab (42.5% of men and 35.2% of women), 121 (35.5%) alirocumab (26.5% of men and 32.8% of women), and 84 (24.6%) inclisiran (26.5% of men and 21.3% of women). Alirocumab at 75 mg was more frequently prescribed to women (10.7% vs. 4.6% in men, *p* = 0.032). This difference appears to be incidental and not driven by specific clinical or demographic factors.

The baseline characteristics of the population are reported in Table 1.

The cohort was followed at four time points: baseline, 341 patients; first follow-up (at 3 and 6 months, respectively, for inclisiran and monoclonal antibodies), 341 patients; second follow-up (at 9 and 12 months, respectively, for inclisiran and monoclonal antibodies), 297 patients; and third follow-up (at 15 and 18 months, respectively, for inclisiran and monoclonal antibodies), 145 patients. The progressive decrease in sample size over time reflects the staggered enrollment and treatment initiation, resulting in variable follow-up durations at the time of analysis.

**Figure 1:**
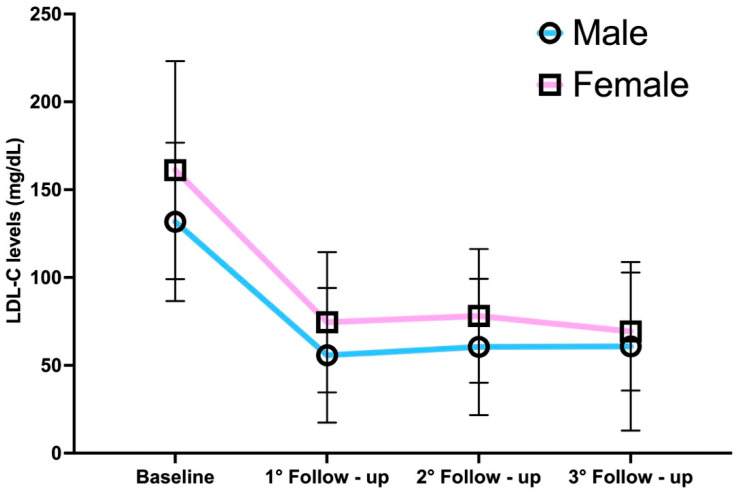
LDL-C levels in women versus men across the follow-ups.

At baseline, LDL-C levels were significantly higher in women than in men (161.1 ± 62.1 mg/dL vs. 131.7 ± 45.1 mg/dL, *p* < 0.001). LDL-C levels decreased markedly following treatment with PCSK9 inhibitors, with sex differences persisting at the first and second follow-ups but disappearing at the third one (Figure 1).

At the first follow-up, a clear sex disparity was observed in percentage reduction, with men showing a 57.3 ± 24.9% reduction compared to 51.7 ± 23.9% in women (*p* = 0.044). However, this difference diminished over subsequent follow-up, stabilizing at approximately 55% at the third follow-up.

At the first follow-up, 41.3% of women and 44.1% of men in the high-risk group reached the LDL-C target of <70 mg/dL, and 63.0% of women and 61.8% of men achieved a ≥50% reduction. At the second follow-up, 32.6% and 39.4%, respectively, reached the target, and 51.5% and 55.8% achieved the ≥50% reduction. At the third follow-up, 35.7% of women and 38.9% of men met the target, while 42.9% and 66.7% had a ≥50% reduction. No significant gender differences were observed in target achievement among high-risk patients.

Among very high risk patients, 67.6% of men and 50.0% of women reached the LDL-C target of <55 mg/dL at the first follow-up (*p* = 0.008), and 68.1% of men and 55.3% of women achieved a ≥50% reduction in cholesterol levels (*p* = 0.049). By the third follow-up, no significant sex differences were found in either target achievement (61.0% of men vs. 64.5% of women, *p* = 0.731) or percentage reduction (*p* = 0.369).

**Figure 2:**
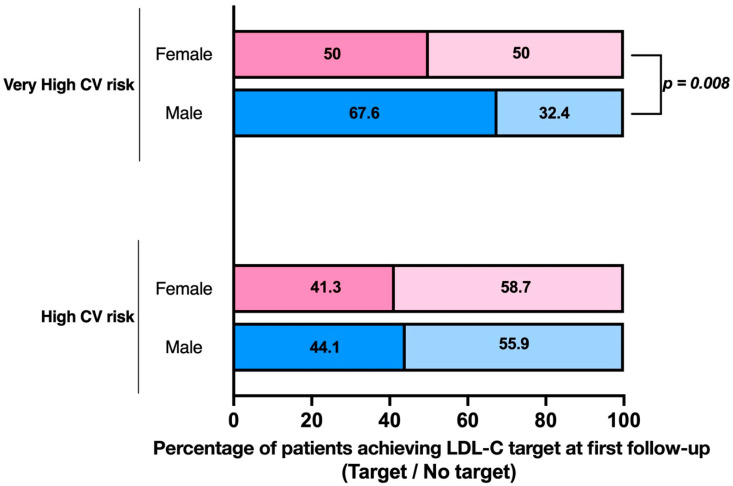
Sex differences in target achievement in very high CV risk versus high-CV risk patients.

Overall adherence to the guidelines was 57.1% at the first follow-up, 52.0% at the second, and 57.5% at the third follow-up. A significant sex difference was observed only at the first control visit, where 44.7% of women compared to 62.2% of men achieved both targets (*p* = 0.010) (Figure 2).

To further explore the potential interaction between sex and type of PCSK9-targeting therapy, we conducted a stratified analysis comparing LDL-C reduction and target attainment between men and women separately for patients treated with monoclonal antibodies (evolocumab or alirocumab) and those treated with inclisiran.

Interestingly, sex-related differences in LDL-C response appeared to be mainly confined to the cohort receiving PCSK9 monoclonal antibodies. Women in this group consistently presented higher baseline LDL-C levels compared to men (163.9 ± 62.7 vs. 133.2 ± 41.5 mg/dL, *p* < 0.001), and this gap persisted through the first and second follow-ups (Table S1; Supplementary Materials), along with significantly lower percentage reductions in LDL-C in women at both time points (52.0% vs. 61.5% at first follow-up, *p* = 0.002; 50.7% vs. 57.5% at second follow-up, *p* = 0.012). These sex-specific differences diminished over time and were no longer statistically significant at the third follow-up (Table S2; Supplementary Materials).

In contrast, no statistically significant sex-based differences were observed in the inclisiran subgroup, either at baseline or at any of the follow-up visits, in terms of either absolute LDL-C levels or percentage reduction (Tables S1 and S2; Supplementary Materials).

A similar pattern was noted for LDL-C-target attainment: in the very high risk group, a significantly lower proportion of women treated with monoclonal antibodies reached the <55 mg/dL goal at the first and second follow-ups compared to men (*p* < 0.001 and *p* = 0.006, respectively), while these differences were not observed in the inclisiran group (Table S4; Supplementary Materials).

The final part of the study aimed to identify potential predictors of LDL-C-target achievement according to the 2019 ESC guidelines [22]. After combining the data from high- and very high cardiovascular risk patients, we first assessed the impact of individual factors through a univariate Poisson regression analysis. Subsequently, we applied multivariate Poisson regression, which simultaneously considered multiple factors. The univariate analysis identified statin intolerance as a negative predictor for both sexes. Sex-specific differences emerged, with HeFH negatively impacting men, while smoking was a significant negative predictor for women. Positive predictors for both sexes included coronary artery disease (CAD), high-intensity statin use, and statin–ezetimibe combination therapy. In men, hypertension and diabetes mellitus were also positive predictors (Table 2).

The multivariate analysis revealed that, in men, only high-intensity statin–ezetimibe use reached statistical significance. In women, statin–ezetimibe and high-intensity statin–ezetimibe therapies were the most significant predictors for achieving LDL-C targets (Table 3).

No significant safety differences were observed according to sex or type of PCSK9 inhibitor. Injection site reactions were the most common adverse events in both men and women treated with evolocumab or alirocumab (4.9% vs. 6.3%, respectively), with mild flu-like symptoms, skin rashes, and musculoskeletal manifestations reported infrequently (3.1% vs. 3.2%, respectively). Seven patients (2.7%), including four men and three women, switched from PCSK9 inhibitors to inclisiran: three (1.1%) due to adverse effects (flu-like symptoms and skin rashes) and four (1.6%) due to low adherence. Also, among patients on inclisiran, injection site reactions were the most frequent adverse event, with no significant sex differences (men: 5.1% vs. women: 3.9%). Other adverse reactions included alopecia in one (3.8%) woman and mild myalgias/arthralgias in one (1.7%) man. Four patients (4.8%) switched back to PCSK9 inhibitors during follow-up due to adverse effects (alopecia, musculoskeletal manifestations, and injection site reactions). In the remaining patients, treatment adherence remained at 100% in both men and women, regardless of therapy.

## 4. Discussion

In this real-world analysis, we evaluated potential gender differences in response to and tolerance of novel PCSK9-targeting therapies. Although the efficacy of these drug classes in improving LDL-C-target achievement is well established, data on gender-specific effects remain limited, especially for inclisiran [23,24,28,29].

When focusing on the female sex, gender emerged as an independent variable associated with a lower likelihood of achieving target LDL-C levels [26,29,30,31,32]. This discrepancy may reflect the longstanding underestimation of CV risk in women and the absence of sex-specific risk factors in traditional scoring systems. Recent ESC guidelines have begun addressing this issue by recognizing female-specific modifiers [33].

Another contributor is physician hesitancy to initiate or escalate high-intensity lipid-lowering therapies in women, potentially due to concerns over adverse effects [19,20,21]. For example, studies have shown a stronger association between the female sex and statin-related side effects, such as statin-associated muscle symptoms (SAMSs); our data reveal a statistical trend related to sex-based differences [25,34,35,36,37,38]. Importantly, female gender remained an independent predictor of failing to reach LDL-C targets, even after adjusting for statin tolerance and background therapy.

PCSK9 inhibitors have been shown to reduce LDL-C levels by up to 60% and are generally well tolerated [29,39,40,41]. However, publications specifically addressing the gender-related impact of PCSK9 inhibitors remain limited [25,26,27,42]. Notably, women tend to have higher LDL-C levels than men, despite receiving the same indication for treatment [43]. While the literature generally reports no gender differences in the prevalence of HeFH [43], our cohort showed that females had higher baseline LDL-C levels than males, primarily due to higher rates of HeFH. Consequently, despite the positive effects of anti-PCSK9 therapies in both sexes, average LDL-C levels in women remained higher than those in men at both the first and second follow-ups. One possible explanation for this disparity is that women may only seek treatment at tertiary centers for lipid disorders when their LDL-C levels are extremely elevated, as seen in familial hypercholesterolemia, leading to lower LDL-C-target achievement despite equivalent treatment. In contrast, men, due to a greater emphasis on risk factor management, are more likely to seek treatment when their LDL-C levels are less elevated. This aligns with recent meta-analyses indicating that there are no gender differences in response to fixed doses of lipid-lowering therapy, yet women with familial hypercholesterolemia (FH) are less likely to receive intensive treatment or meet guideline-recommended LDL-C targets [44].

Another open question, given the limited number of publications on this matter, is whether there might be a different therapeutic response between the sexes to PCSK9 inhibitors. A recent real-world registry study revealed that the addition of PCSK9mAb to maximum tolerated lipid-lowering therapy resulted in a smaller reduction in LDL-C levels among women compared to men at the 6-month follow-up, which was not solely attributed to higher baseline LDL-C levels in women [26]. We did not observe statistically significant gender differences regarding the percentage reduction in LDL-C at 12 months of treatment. However, due to higher baseline LDL-C levels, fewer women compared to men showed adherence to the 2019 ESC guidelines, regardless of whether they were receiving background lipid-lowering therapy. These data suggest that the overall efficacy of PCSK9 inhibitors is comparable between sexes. A trend toward a slower LDL-C reduction in women was observed during early follow-up visits; however, this should be interpreted with caution, as a formal time-to-response analysis was not performed. Notably, most studies on PCSK9 inhibitors have shorter follow-up durations than ours, which may limit the observation of temporal response patterns between sexes [26,45,46].

Additional stratified analyses support the hypothesis that the delayed LDL-C response in women is primarily observed in patients treated with PCSK9 monoclonal antibodies. In contrast, no significant sex-based differences were found among patients receiving inclisiran. This observation is consistent with the limited available data, although current evidence remains insufficient to draw definitive conclusions [26].

Overall, the combination of PCSK9 inhibitors with high-intensity statins significantly improved outcomes across both sexes, reinforcing the importance of combination therapy in achieving LDL-C goals [29,39,40].

Finally, treatment tolerance was excellent across both groups, with few adverse reactions and high adherence. Most side effects were mild and limited to injection-site reactions. These findings are consistent with previous real-world studies highlighting the favorable safety profile and persistence of PCSK9 inhibitors in both men and women [47].

### Strengths and Limitations

Our study has several strengths, including its real-world, prospective design and long-term follow-up of patients initiating PCSK9 inhibitor therapy. It is also among the first to include inclisiran-treated patients, thus contributing to the limited existing evidence on this newer agent.

However, some limitations must be acknowledged. The observational and monocentric design introduces potential selection and confounding biases, and the overall sample size—although comparable to similar studies—remains relatively small. The cohort was largely composed of patients with suspected HeFH or statin intolerance, which may limit the generalizability of our findings. Moreover, the unequal sex distribution likely reflects real-world referral patterns and could affect sex-specific analyses.

A key limitation is the significantly higher baseline LDL-C levels and HeFH prevalence among women. Although these variables were accounted for in multivariate sex-specific models, residual confounding cannot be ruled out without more advanced causal inference approaches.

An additional concern is the small number of patients treated with inclisiran. Once stratified by sex and cardiovascular risk, subgroup sizes became especially limited, reducing the statistical power and increasing the risk of selection bias. As such, any findings regarding sex-specific differences in response to inclisiran should be interpreted with caution and considered exploratory.

Finally, while monoclonal antibodies were self-administered, adherence was not formally monitored, which may have influenced LDL-C outcomes. Moreover, no time-to-response analysis was performed, and the limited number of follow-up assessments may have constrained the evaluation of response dynamics over time.

## 5. Conclusions

This real-world study confirms that PCSK9 inhibitors are effective in both sexes. The slightly attenuated LDL-C reduction observed in women at earlier follow-up points may reflect a slower onset of response specifically to monoclonal antibody PCSK9 inhibitors, although this should be interpreted cautiously given the limited follow-up and absence of a formal time-to-response analysis. While both sexes benefited from lipid-lowering effects, fewer women reached LDL-C targets, likely due to higher baseline levels, possibly linked to higher rates of familial hypercholesterolemia. However, although the overall prevalence of ASCVD is similar between men and women, female patients remain under-represented among very high risk individuals and are less frequently referred to specialized lipid clinics. This may be due to underestimation of cardiovascular risk in women, atypical symptom presentation, or lower referral and diagnosis rates, ultimately resulting in less intensive treatment and follow-up.

## Figures and Tables

**Table 1:** Demographic and baseline clinical characteristics stratified by cardiovascular (CV) risk class.

Variable		Total (*n* = 341)	Men (*n* = 219)	Women (*n* = 122)	*p*
**Demographics**	*Age (years), average ± SD*	64.8 ± 12.1	63.9 ± 11.8	66.4 ± 12.6	0.072
**CV risk**	*High CV risk, n (%)*	80 (23.5)	34 (15.5)	46 (37.7)	**<0.001**
	*Very high CV risk, n (%)*	261 (76.5)	185 (84.5)	76 (62.3)	**<0.001**
**Type of dyslipidemia**	*HeFH, n (%)*	159 (46.6)	84 (38.4)	75 (61.5)	**<0.001**
	*Non-familial dyslipidemia, n (%)*	182 (53.4)	135 (61.6)	47 (38.5)	**<0.001**
**Cardiovascular risk factors**	*Smokers, n (%)*	51 (15.0)	28 (12.8)	23 (18.9)	0.133
	*Former smokers, n (%)*	141 (41.3)	103 (47.0)	38 (31.1)	**0.004**
	*Hypertension, n (%)*	206 (60.4)	135 (61.6)	71 (58.2)	0.533
	*T2DM, n (%)*	43 (12.6)	27 (12.3)	16 (13.1)	0.834
	*Family history of ASCVD* *, n (%)*	151 (44.4)	103 (47.2)	48 (39.3)	0.160
	*Hyperuricemia, n (%)*	10 (2.9)	9 (4.1)	1 (0.8)	0.083
**ASCVD**	*CAD, n (%)*	214 (62.8)	165 (75.3)	49 (40.2)	**<0.001**
	*PCI, n (%)*	183 (53.7)	143 (65.3)	40 (32.8)	**<0.001**
	*CABG, n (%)*	30 (8.8)	22 (10.0)	8 (6.6)	0.276
	*Ictus/TIA, n (%)*	25 (7.3)	14 (6.4)	11 (9.0)	0.374
	*PAD, n (%)*	78 (22.9)	47 (21.5)	31 (25.4)	0.406
	*Peripheral revascularization, n (%)*	33 (9.7)	23 (10.5)	10 (8.2)	0.491
**Comorbidities**	*Heart failure, n (%)*	13 (3.8)	10 (4.6)	3 (2.5)	0.331
	*Chronic kidney disease, n (%)*	10 (2.9)	6 (2.7)	4 (3.3)	0.778
**Lipid-lowering therapy**	*None, n (%),*	58 (17.0)	31 (14.2)	27 (22.1)	0.061
	*Statin only, n (%)*	18 (5.3)	12 (5.5)	6 (4.9)	0.824
	*High-intensity statin only, n (%)*	15 (4.4)	10 (4.6)	5 (4.1)	0.840
	*Moderate-intensity statin only, n (%)*	3 (0.9)	2 (0.9)	1 (0.8)	0.929
	*Low-intensity statin only, n (%)*	0 (0.0)	0 (0.0)	0 (0.0)	1.000
	*Ezetimibe only, n (%)*	100 (29.3)	61 (27.9)	39 (32.0)	0.425
	*Statin + ezetimibe, n (%)*	165 (4.4)	115 (52.5)	50 (41.0)	**0.041**
	*High-intensity statin + ezetimibe, n (%)*	119 (34.9)	82 (37.4)	37 (30.3)	0.187
	*Moderate-intensity statin + ezetimibe, n (%)*	40 (11.7)	29 (13.2)	11 (9.0)	0.246
	*Low-intensity statin + ezetimibe, n (%)*	6 (1.76)	4 (1.8)	2 (1.6)	0.900
	*Bempedoic acid, n (%)*	9 (2.6)	7 (3.2)	2 (1.6)	0.391
	*Statin intolerance, n (%)*	164 (48.1)	96 (43.8)	68 (55.7)	0.035
**PCSK9i**	*Evolocumab 140 mg, n (%)*	136 (39.9)	93 (42.5)	43 (35.2)	0.192
	*Alirocumab 150 mg, n (%)*	98 (28.7)	58 (26.5)	40 (32.8)	0.218
	*Alirocumab 75 mg, n (%)*	23 (6.7)	10 (4.6)	13 (10.7)	**0.032**
	*Inclisiran, n (%)*	84 (24.6)	58 (26.5)	26 (21.3)	0.289

SD: standard deviation; CV: cardiovascular; T2DM: type 2 diabetes mellitus; HeFH: heterozygous familial hypercholesterolemia; ASCVD: atherosclerotic cardiovascular disease; CAD: coronary artery disease; PCI: percutaneous coronary intervention: CABG: coronary artery bypass graft surgery; TIA: transient ischemic attack; PAD: peripheral artery disease; PCSK9i: proprotein convertase subtilisin/kexin type 9 inhibitor. Bold values indicate statistically significant *p*-values (*p* < 0.05).

**Table 2:** Univariate Poisson regression analysis for LDL-C-target-level attainment stratified for sex.

Variable	Women			Men		
	**IRR**	**95% C.I.**	***p*-Value**	**IRR**	**95% C.I.**	***p*-Value**
**Smoker**	**3.010**	**(8.271–1.096)**	**0.033**	0.819	(1.908–0.351)	0.643
**Former smoker**	0.826	(1.780–0.383)	0.626	1.158	(2.012–0.666)	0.603
**Hypertension**	0.894	(1.840–0.434)	0.761	**0.528**	**(0.929–0.300)**	**0.027**
**T2DM**	1.148	(3.309–0.398)	0.798	**0.362**	**(0.999–0.132)**	**0.050**
**Family history of ASCVD**	0.805	(1.667–0.389)	0.559	0.900	(1.565–0.517)	0.708
**CAD**	**0.277**	**(0.592–0.129)**	**0.001**	**0.415**	**(0.778–0.222)**	**0.006**
**Stroke/TIA**	1.058	(3.669–0.305)	0.930	0.277	(1.271–0.060)	0.099
**PAD**	1.859	(4.317–0.800)	0.149	1.005	(1.968–0.514)	0.988
**Peripheral revascularization**	0.867	(3.161–0.238)	0.828	0.458	(1.285–0.163)	0.138
**HeFH**	1.006	(2.089–0.484)	0.988	**1.894**	**(3.332–1.077)**	**0.027**
**Statin intolerance**	**23.326**	**(7.018–1.576)**	**0.002**	**2.310**	**(4.055–1.316)**	**0.004**
**At least one LTT**	**0.279**	**(0.687–0.113)**	**0.005**	**0.431**	**(0.938–0.198)**	**0.034**
**Statin + ezetimibe**	1.333	(3.400–0.523)	0.547	0.546	(1.229–0.242)	0.144
**High-dose statin**	**0.146**	**(0.394–0.054)**	**<0.001**	**0.362**	**(0.698–0.188)**	**0.002**
**High-dose statin + ezetimibe**	**0.162**	**(0.472–0.056)**	**0.001**	**0.333**	**(0.674–0.164)**	**0.002**

T2DM: type 2 diabetes mellitus; ASCVD: atherosclerotic cardiovascular disease; CAD: coronary artery disease; TIA: transient ischemic attack; PAD: peripheral artery disease; HeFH: heterozygous familial hypercholesterolemia; LLT: lipid-lowering therapy. Bold values indicate statistically significant *p*-values (*p* < 0.05).

**Table 3:** Multivariate Poisson regression analysis for LDL-C-target-level attainment stratified for sex.

	**Women**		
**Variable**	**IRR**	**95% C.I.**	***p*-Value**
**Smoker** **At least one LTT**	**3.418** **0.259**	**(10.839–1.078)** **(0.820–0.082)**	**0.037** **0.022**
**Statin + ezetimibe**	4.804	(24.668–0.936)	0.060
**High-dose statin + ezetimibe**	**0.048**	**(0.268–0.008)**	**0.001**
	**Men**		
**Variable**	**IRR**	**95% C.I.**	***p*-value**
**Former smoker**	1.723	(3.239–0.917)	0.091
**Hypertension**	**0.499**	**(0.940–0.265)**	**0.031**
**CAD** **Stroke/TIA**	**0.469** **0.196**	**(0.930–0.236)** **(0.947–0.0419**	**0.030** **0.043**
**High-dose statin + ezetimibe**	**0.334**	**(0.642–0.173)**	**0.001**

CAD: coronary artery disease; TIA: transient ischemic attack. Bold values indicate statistically significant *p*-values (*p* < 0.05).

## Data Availability

The data presented in this study are not publicly available due to privacy and ethical restrictions, as they derive from clinical research involving human participants. However, anonymized datasets may be made available from the corresponding author upon reasonable request and subject to approval by the appropriate ethics committee.

## Supplementary Materials

The following supporting information can be downloaded at https://www.mdpi.com/article/10.3390/jcm14134469/s1: Tables S1–S4: Sex-Stratified Analysis by type of PCSK9-targeting therapy: Mean LDL-C levels by sex and treatment type over time (S1); Percentage reduction in LDL-C by sex and treatment type over time (S2); Percentage of patients achieving LDL-C targets with PCSK9 monoclonal antibodies, stratified by sex and cardiovascular risk category (S3); Percentage of patients achieving LDL-C targets with inclisiran, stratified by sex and cardiovascular risk category (S4).
