# Supplementary material for: PCSK9-Targeting Drugs and Gender: Are There Any Differences?
Source: J Clin Med. 2025 Jun 24;14(13):4469. doi: 10.3390/jcm14134469 (PMC12250220; doi:10.3390/jcm14134469)
Supplement: Supplementary file 1 [file jcm-14-04469-s001.zip › jcm-3654040-supplementary.pdf]

SUPPLEMENTARY MATERIALS

Sex-Stratified Analysis by Type of PCSK9-Targeting Therapy

LDL-C Mean Levels

PCSK9 ab				
**Visit**	**Overall, mean±SD (*n*=257)**	**Male, mean±SD (*n*= 161)**	**Female, mean±SD (*n*=96)**	***p***
Baseline	144.7±52.5	133.2±41.5	163.9±62.7	**<0.001**
First follow-up	59.5±37.5	50.8±34.5	73.9±38.1	**<0.001**
Second follow-up	63.5±38.9	56.3±38.1	75.9±37.2	**<0.001**
Third follow-up	61.4±41.8	57.7±45.7	68.6±31.8	**0.008**
**Inclisiran**				
**Visit**	**Overall, mean±SD (*n*= 84)**	**Male, mean±SD (*n*=58)**	**Female, mean±SD (*n*=26)**	***p***
Baseline	134.4±56.5	127.5±54.0	150.2±59.7	0.108
First follow-up	71.5±45.3	69.3±44.7	76.5±47.0	0.498
Second follow-up	82.4±38.4	78.7±37.1	90.81±41.4	0.346
Third follow-up	75.9±52.1	77.73±57.5	72.50±43.6	0.821

Table S1. Mean LDL-C levels by sex and treatment type over time.
Mean LDL-C concentrations (mg/dL) at baseline and across follow-up visits are shown for patients treated with PCSK9 monoclonal antibodies and inclisiran, stratified by sex. A significant sex-based difference was observed in the monoclonal antibody group, with consistently higher LDL-C levels in women at all time points, while no significant differences were found in the inclisiran group.

Percentage reduction in LDL-C

PCSK9 ab				
**Visit**	**Overall, mean±SD (*n*=257)**	**Male, mean±SD (*n*= 161)**	**Female, mean±SD (*n*=96)**	***p***
First follow-up	57.9±24.4	61.5±23.4	52.0±25.0	**0.002**
Second follow-up	55.0±25.2	57.5±25.5	50.7±24.0	**0.012**
Third follow-up	57.1±26.2	57.9±29.0	55.3±19.4	0.120
**Inclisiran**				
**Visit**	**Overall, mean±SD (*n*= 84)**	**Male, mean±SD (*n*=58)**	**Female, mean±SD (*n*=26)**	***p***
First follow-up	47.3±24.0	45.8±25.7	50.5±19.9	0.405
Second follow-up	39.7±22.9	37.6±24.1	44.6±19.7	0.351
Third follow-up	46.9±21.7	43.3±18.4	53.8±26.7	0.121

Table S2. Percentage reduction in LDL-C by sex and treatment type over time.
Mean percentage reductions in LDL-C from baseline at each follow-up visit are reported for both treatment groups, stratified by sex. In patients receiving PCSK9 monoclonal antibodies, women showed significantly lower LDL-C reductions at the first and second follow-ups. No statistically significant sex-based differences were observed among inclisiran-treated patients.

Percentage of patients at target

**PCSK9 ab**				
**High CV Risk (<70)**				
**Visit**	**Overall, *n* (%) (*n*= 56)**	**Male, *n* (%) (*n*=20)**	**Female, *n* (%) (*n*= 36)**	***p***
First follow-up	27 (48.2)	8 (40.0)	19 (52.8)	0.364
Second follow-up	24 (43.6)	10 (50.0)	14 (40.0)	0.476
Third follow-up	11 (45.8)	4 (44.4)	7 (46.7)	0.918
**Very High CV Risk (<55)**				
**Visit**	**Overall, *n* (%) (*n*=201)**	**Male, *n* (%) (*n*=141)**	**Female, *n* (%) (*n*=60)**	***p***
First follow-up	71 (35.3)	39 (27.7)	32 (53.3)	**<0,001**
Second follow-up	109 (57.4)	86 (63.7)	23 (41.8)	**0,006**
Third follow-up	62 (63.3)	46 (63.9)	16 (61.5)	0.832

Table S3. Percentage of patients achieving LDL-C targets with PCSK9 monoclonal antibodies, stratified by sex and cardiovascular risk category.
Proportion of male and female patients treated with evolocumab or alirocumab who achieved guideline-recommended LDL-C targets (<70 mg/dL for high CV-risk, <55 mg/dL for very-high CV-risk) at each follow-up visit. In the very high-risk group, significantly fewer women achieved the <55 mg/dL goal at early follow-ups compared to men.

**Inclisiran**				
**High CV-Risk (<70)**				
**Visit**	**Overall, *n* (%) (*n*=24)**	**Male, *n* (%) (*n*=14)**	**Female, *n* (%) (*n*=10)**	***p***
First follow-up	19 (79.2)	11 (78.6)	8 (80.0)	0.934
Second follow-up	3 (14.3)	3 (23.1)	0 (0.0)	0.152
Third follow-up	1 (12.5)	1 (20.0)	0 (0)	0.439
**Very High CV-Risk (<55)**				
**Visit**	**Overall, *n* (%) (*n*=60)**	**Male, *n* (%) (*n*=44)**	**Female, *n* (%) (*n*= 16)**	***p***
First follow-up	27 (45.0)	21 (47.7)	6 (37.5)	0.485
Second follow-up	15 (48.4)	11 (47.8)	4 (50.0)	0.917
Third follow-up	8 (53.3)	4 (40.0)	4 (80.0)	0.157

Table S4. Percentage of patients achieving LDL-C targets with inclisiran, stratified by sex and cardiovascular risk category.

Proportion of male and female patients treated with inclisiran who achieved LDL-C targets according to cardiovascular risk class at each follow-up visit. No statistically significant sex-based differences were observed, although the small sample size, particularly in later follow-ups, limits interpretability.
